# Evaluation of Yeast Strains for Pomegranate Alcoholic Beverage Production: Effect on Physicochemical Characteristics, Antioxidant Activity, and Aroma Compounds

**DOI:** 10.3390/microorganisms8101583

**Published:** 2020-10-14

**Authors:** Evangelos Kokkinomagoulos, Anastasios Nikolaou, Yiannis Kourkoutas, Panagiotis Kandylis

**Affiliations:** 1 Laboratory of Oenology and Alcoholic Beverages, Department of Food Science and Technology, School of Agriculture, Aristotle University of Thessaloniki, P.O. Box 235, 54124 Thessaloniki, Greece; ekokkinom@gmail.com; 2 Laboratory of Applied Microbiology & Biotechnology, Department of Molecular Biology & Genetics, Democritus University of Thrace, GR-68100 Alexandroupolis, Greece; anikol@mbg.duth.gr (A.N.); ikourkou@mbg.duth.gr (Y.K.)

**Keywords:** pomegranate, alcoholic fermentation, antioxidants, phenolic compounds, volatile profile, GC/MS, flavonoid, *Punica granatum* L.

## Abstract

In the present study, three commercial yeasts (for wine, beer, and cider) were evaluated for the production of pomegranate alcoholic beverage (PAB) from a juice of Wonderful variety. The physicochemical characteristics, antioxidant activity, and aromatic profiles of PABs were investigated before and after fermentation, while the effect of yeast strain and fermentation temperature (15 and 25 °C) was also evaluated. The PABs contained ethanol in the ranges of 5.6–7.0% v/v, in combination with glycerol (2.65–6.05 g L^−1^), and low volatile acidity. Total flavonoid content, total phenolic content, free radical-scavenging activity, and total monomeric anthocyanin content appeared to decrease after fermentation, possibly due to hydrolysis, oxidation, and other reactions. In general, PABs retained 81–91% of free radical-scavenging activity, 29–41% of phenolics, 24–55% of flavonoids, and 66–75% of anthocyanins. The use of different yeast affected mainly flavonoids and anthocyanins, and yeast strain M02 resulted in the highest values after fermentation. In PABs, 30 different volatile compounds were identified, specifically 15 esters, 4 organic acids, 8 alcohols, and 3 terpenes. The principal component analysis showed that the fermentation temperature affected significantly volatile composition, whereas, among the yeasts, WB06 is the one that seems to differentiate. The findings of this study show that the selection of the appropriate yeast and fermentation temperature is very crucial and affects the characteristics of the final product.

## 1. Introduction

Pomegranate (*Punica granatum* L.) is a fruit that has aroused scientific interest for decades, due to the numerous beneficial effects that it can possess as food, its pharmacological and toxicological properties, such as antioxidant, antimicrobial, anti-inflammatory, and anticarcinogenic, and numerous applications in several food products [1]. Pomegranate is a well-known source of valuable nutrients, whereas the production and consumption are showing continuously increasing trends worldwide, a fact that can be probably related with the increase of consumers’ awareness regarding the possible health benefits of pomegranate consumption, through the development of science and technology. Additionally, pomegranate consumption is not restricted only to fresh fruit, but it also exists in the market as another alternative product, as pomegranate wine or pomegranate alcoholic beverage (PAB), a product of alcoholic fermentation.

It is known that fruit processing (e.g., pasteurization) can lead to a reduction of fresh fruit aromatic intensity, which in the case of pomegranate, is already low. Consequently, these processes can influence consumer acceptance. The fact that pomegranates generally possess low aromatic intensities means that the isolation and identification of volatile aromatic compounds have been difficult, whereas different studies suggest different results regarding the aromatic profile of pomegranate and its products. According to the literature, hexanal, limonene, trans-2-hexanal, cis-2-hexanal, and a-terpineol are the major aromatic compounds of fresh pomegranate fruit [2], while 3-methyl butanal, ethyl butanoate, isopentyl acetate, hexanol, diethyl allyl malonate, and α-ionone in the case of pomegranate juice [3]. Studies regarding the aroma of PABs are limited, and they reported ethyl octanoate, ethyl decanoate, ethanol, 3-methyl−1-butanol, phenylethyl alcohol, and 3-methyl−1-butanol acetate, as main flavor compounds [4].

Since PAB is a fermentation product, its main organoleptic characteristics are affected to a large extent by fermentation and especially by the microorganisms used, as was also proved in several fermented beverages made from red dragon fruit [5], diluted honey [6], Pilsner wort [7], apple juice [8] and grape juice [9]. Several studies reported that fermentation of pomegranate juice resulted in a completely new product with altered bioactive components, mainly due to reactions of polymerization, condensation, oxidation, hydrolysis, enzyme activity, and interactions of antioxidants with yeast cell walls [10]. Nevertheless, to the best of our knowledge, the effect of different yeasts on flavor characteristics and particular bioactive compounds in PAB has not been well investigated, and it is only limited in studies with one yeast strain [4,11].

The main objectives of the present study were to investigate the physicochemical characteristics, antioxidant properties, bioactive compounds, and flavor profile of PABs fermented with three commercial yeast strains at 15 °C and 25 °C. Total phenolic content (TPC), total flavonoid content (TFC), DPPH^●^ free radical, main physicochemical characteristics like pH, acidity (total and volatile), ethanol content, and reducing sugars, as well as flavor profiles were monitored before and after the fermentation process. To the best of our knowledge, this is the first time to evaluate the combined effect of yeast strain and fermentation temperature on the characteristics of PAB.

## 2. Materials and Methods

### 2.1. Pomegranate Juice and Yeast Strains

Commercial pasteurized 100% natural pomegranate (cv. Wonderful) juice (En Karpo, Oraiokastro, Greece) was used in this study. Three commercial yeasts were employed: Craft Series SN9 Wine yeast (*Saccharomyces bayanus*), M02-Cider (*Saccharomyces cerevisiae*) (Mangrove Jack’s, Albany, Auckland, New Zealand), and SAFALE™ WB-06 (*Saccharomyces cerevisiae* var. *diastaticus*) (Fermentis by Lesaffre, Marcq-en-Barœul, France).

### 2.2. Fermentation of Pomegranate Juice

Yeasts were activated prior to inoculation through rehydration in sterile distilled water and were added to 200 mL of pomegranate juice according to the manufacturers’ directions (resulting in: M02 3.6 x 10^5^ CFU mL^−1^, SN9 2.5 x 10^5^ CFU mL^−1,^ and WB06 2.4 x 10^7^ CFU mL^−1^). Fermentations were carried out in sterile glass containers, with airlocks containing 70% v/v ethanol solution applied to them. Fermentations were carried out at 15 °C and 25 °C. All fermentations were carried out in triplicate, and their progress was monitored by determining the weight loss as a result of CO_2_ production and release. The weight loss was monitored until reaching a stable weight [12].

### 2.3. Microbiological Analysis

After inoculation, all samples were analyzed microbiologically in order to confirm the initial inoculum. Specifically, 1 mL of inoculated pomegranate juice was sampled, and serial dilutions were performed in peptone water (1 g L^−1^ peptone, 8.5 g L^−1^ sodium chloride). Appropriate dilutions were plated on a Petri dish with YPD (Yeast extract Peptone Dextrose) agar (10 g L^−1^ yeast extract, 20 g L^−1^ peptone, 20 g L^−1^ glucose, 30 g L^−1^ bacteriological agar) and were incubated at 30 °C for 3–5 days. Results were expressed as CFU (Colony Forming Units) mL^−1^.

### 2.4. Determination of Reducing Sugars

Reducing sugars (RS) were determined using the DNS (3,5-dinitrosalicylic acid) method [13], with some modifications. Specifically, in a glass test tube, 500 μL of sample (appropriately diluted with distilled water) and 500 μL of DNS solution (10 g L^−1^ 3,5-dinitrosalicylic acid, 300 g L^−1^ potassium sodium tartrate, 16 g L^−1^ NaOH) were added, vortexed, and placed at 100 °C for 5 min. The tubes were then cooled to room temperature and 5 mL of distilled water were added. The tubes were vortexed, and absorbance was recorded at 540 nm in a UV/Vis spectrophotometer (UV−1800, Shimadzu, Kyoto, Japan) and quantified using D-glucose as a standard.

### 2.5. Determination of Ethanol and Glycerol Content

Ethanol and glycerol were determined by an HPLC Shimadzu chromatography system (Shimadzu Corp., Germany) equipped with a Nucleogel ION 300 OA column (Macherey-Nagel, Germany) [14].

### 2.6. pH, Volatile Acidity, and Total Acidity

The pH values were determined by portable, electronic pH-meter (SensoDirect pH 110, AQUALYTIC, Dortmund, Germany). Total and volatile acidities were determined using the OIV-MA-AS313-01 and OIV-MA-AS313-02 methods, respectively [15].

### 2.7. Determination of Total Flavonoid Content

Total flavonoid content (TFC) was determined by the flavonoid-aluminum chloride (AlCl_3_) complexation spectrophotometric method [16], with some modifications. Specifically, in a 5-mL Eppendorf tube, 2 mL of sample (appropriately diluted with distilled water), 200 μL of AlCl_3_ solution (20 g L^−1^ AlCl_3_ in methanolic solution of 50 mL L^−1^ acetic acid), and 2.8 mL of 50 mL L^−1^ acetic acid in methanol were added, vortexed and allowed to stand at room temperature for 30 min. The absorbance was recorded at 415 nm in a UV/Vis spectrophotometer (UV−1800, Shimadzu, Kyoto, Japan) and quantified using quercetin as a standard.

### 2.8. Determination of Total Phenolic Content

Total phenolic content (TPC) was determined by the Folin-Ciocalteau method [17], with some modifications. Specifically, in a 5-mL Eppendorf tube, 3.95 mL of distilled water, 50 μL of sample (appropriately diluted with distilled water), and 250 μL of Folin-Ciocalteau reagent were added and vortexed. After 1 min, 750 μL of 200 g L^−1^ sodium carbonate were added, vortexed, and allowed to stand at room temperature in darkness, for 120 min. The absorbance was recorded at 750 nm in a UV/Vis spectrophotometer (UV−1800, Shimadzu, Kyoto, Japan) and quantified using gallic acid as a standard.

### 2.9. Determination of Free Radical-Scavenging Activity

Free radical-scavenging activity was determined using the free radical DPPH^●^ (2,2 diphenyl−1-picrylhydrazyl) method [17], with some modifications. Specifically, in a 5-mL Eppendorf microtube, 125 μL of sample (appropriately diluted with distilled water) and 4.875 mL of DPPH^●^ solution were added, vortexed and allowed to stand at room temperature in darkness, for 30 min. The absorbance of samples and blank (no sample) were recorded at 515 nm in a UV/Vis spectrophotometer (UV−1800, Shimadzu, Kyoto, Japan) and quantified using Trolox as a standard.

### 2.10. Determination of Total Monomeric Anthocyanin Content

Total monomeric anthocyanin content (TMAC) was determined using the pH differential method [18], with some modifications. Specifically, in a 5-mL Eppendorf tube, 5 mL of sample (appropriately diluted with 0.025 Μ potassium chloride buffer (pH 1.0)) was added, vortexed, and allowed to stand at room temperature for 20 min. Additionally, in a 5-mL Eppendorf tube, 5 mL of sample (appropriately diluted with 0.4 M sodium acetate buffer (pH 4.5)) was added, vortexed, and allowed to stand at room temperature for 20 min. The absorbance was recorded at 520 and 700 nm in a UV/Vis spectrophotometer (UV−1800, Shimadzu, Kyoto, Japan). Results were expressed as mg cyanidin-3-glucoside equivalents (Cy3GE) L^−1^ according to the following equation: $cyanidin-3-glucoside\;\left(mg\;L^{-1}\right)=\;\frac{A*MW*DF*10^{3}}{\varepsilon *1},$ where A = (A_520nm—_A_700nm_) pH 1.0—(A_520nm_-A_700nm_) pH 4.5; MW (Molecular Weight) = 449.2 g mol^−1^ for cyanidin-3-glucoside, DF (Dilution Factor), 10^3^ = factor for conversion from g to mg and ε (molar extinction coefficient) = 26900 L mol^−1^ cm^−1^ for cyanidin-3-glucoside.

### 2.11. Determination of Yeast Assimilable Nitrogen

Yeast assimilable nitrogen (YAN) was determined following the formol method [19].

### 2.12. HS-SPME GC/MS Analysis 

Pomegranate alcoholic beverage samples from fermentations carried out at 15 and 25 °C were subjected to headspace solid-phase microextraction (HS-SPME) GC/MS analysis using a GC/MS (6890N GC, 5973 NetworkedMS MSD, Agilent Technologies, Santa Clara, CA, USA) equipped with a HP-5MS column (30 m, 0.25 mm i.d., 0.25 μm film thickness, Agilent Technologies, Santa Clara, CA, USA) as recently described [20].

### 2.13. Odor Activity Value (OAV)

The OAV was calculated as the ratio between the concentration of an individual compound (detected in the present study) and the perception threshold found in the literature.

### 2.14. Statistical Analysis

The results were analyzed statistically by ANOVA. Bonferroni *post-hoc* test was used to determine significant differences among results (Statistica version 12.0, StatSoft Inc., Tulsa, OK, USA). The principal component analysis (PCA) algorithm was computed using XLSTAT 2015.1 [14].

## 3. Results and Discussion

In the present study, commercial pomegranate juice (sugars 128.4 ± 0.1 g L^−1^; pH 3.12 ± 0.01; acidity 16.0 ± 0.1 g citric acid L^−1^; YAN 146.6 ± 4.4 mg nitrogen L^−1^) was used for the production of PABs after fermentation with different yeast strains. There is no specific yeast strain designed for pomegranate fermentation and therefore, the selection of appropriate yeast is very crucial. In order to deal with this gap in the literature, the aim of the present study was to evaluate different commercial yeast strains that are usually used in well-known fermented products like wine, beer, and cider and select the most appropriate for pomegranate fermentation. In addition, two fermentation temperatures, 15 and 25 °C were selected, in order to evaluate each yeast performance. These temperatures are the usual used in winemaking (near 15 °C for white wines and near 25 °C for red wines), and they have been proved ideal to compare the volatile profile of different yeasts [21,22]. Fermentations near 15 °C benefit the sensory quality of wines since yeasts produce several alcohols and acetates and the loss of aroma is minimized [23], while 25 °C favors the growth of *S. cerevisiae* [24].

### 3.1. Physicochemical Characteristics

**Figure 1:**
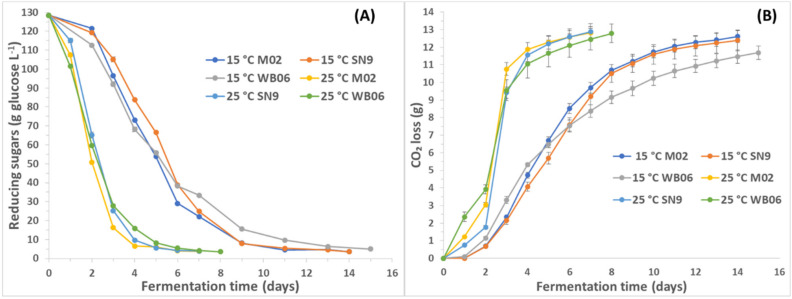
Effect of yeast strain and temperature on fermentation kinetics (reducing sugars (**A**) and CO_2_ loss (**B**)) of PABs.

The fermentation behavior of each yeast strain is presented in Figure 1, where sugar consumption and CO_2_ release are reported during fermentation. No significant differences are reported apart from a slight delay in the completion of fermentation by WB06.

Table 1 summarizes the effects of yeast and fermentation temperature on the physicochemical characteristics of PABs, in comparison with the characteristics of the initial pomegranate juice.

Higher amounts of residual sugars were detected in PAB with WB06 yeast at both temperatures. It can also be deduced that at 15 °C yeasts consumed less sugars in comparison with 25 °C, a fact that can be due to the temperature stress that yeasts underwent. However, all effects seemed to be significant (*p* <0.05), while it appeared that the effect of temperature was more significant for yeasts M02 and WB06.

Ethanol content was affected significantly by yeasts but not by temperature. Also, a combined effect of yeast and temperature was reported. Alcohol concentrations are not in full accordance with other studies, as Andreu-Sevilla et al. [11] observed final concentrations of 8.30–9.05% v/v, while in the present study, the values were lower (5.6–7.0% v/v). However, this characteristic is tightly related with the initial sugar content of the juice. Moreover, the effect of yeast appeared to be significant at 15 °C, as fermentations with WB06 led to significantly lower concentrations, while at 25 °C no significant differences were observed (*p* > 0.05).

Glycerol is a secondary product of *Saccharomyces cerevisiae* fermentation, a non-volatile alcohol that exclusively affects taste and texture, as it increases sweetness and fullness [25]. All yeasts examined presented a similar pattern of glycerol production in fermentations at 25 °C, whereas all findings were significantly higher in comparison with other studies [26]. This pattern is confirmed statistically, revealing the significant effect of temperature, yeast, and both of them. Additionally, it appears that the effect of temperature was not significant (*p* >0.05) only in the case of fermentation with M02, as a similar amount of glycerol was produced at both temperatures. Similar concentrations of glycerol (4.4–5.2 g L^−1^), produced by *S. cerevisiae*, have been reported in an alcoholic beverage from dragon fruit [5], however, this yeast is capable of producing even higher amounts in the case of mead fermentation (>9.5 g L^−1^) [6].

A crucial factor for all types of fermentation is pH because it affects the growth of yeasts and characteristics of the final product, such as color and taste. The pH range of pomegranate juice is 2.9–3.7 and varies according to different pomegranate variety [26,27,28,29,30]. In the present study, the pH value of the juice used was 3.12, which is similar with other studies with cv Wonderful, reporting the usual pH values between 2.9 and 3.2 [27,29]. After fermentation, a slight reduction in pH was observed, especially at 25 °C resulting in a final pH value of 3.10–3.12 at 15 °C and 3.05–3.07 at 25 °C. Similar pH values (3.12) have been observed on fermented beverages from Wonderful variety in previous studies [29]. Fermentations at 15 °C did not alter pH significantly, in contrast with fermentations at 25 °C. In conclusion, the effect of yeast was not significant at both temperatures, and only temperature significantly affected the pH values.

Fermentations with SN9 yeast led to the highest volatile acidity (0.83–0.88 g acetic acid L^−1^), followed by M02 yeast (0.71–0.76 g acetic acid L^−1^) and WB06 yeast (0.22–0.33 g acetic acid L^−1^). However, all final products comply with the regulations concerning volatile acidity (≤1 g acetic acid L^−1^, of the Greek Government [31]), whereas other studies present slightly higher volatile acidities (0.9–1.0 g acetic acid L^−1^) [28]. In general, the results showed that volatile acidity is a characteristic that it is mainly affected by the yeast used for the fermentation, and to a lesser extent, by the fermentation temperature.

Total acidity did not increase significantly after fermentation, with the higher value presented at fermentations at 25 °C (M02, 17.0 ± 0.2 g citric acid L^−1^). Similarly, all final products complied with the regulations of the Greek Government [31] concerning total acidity (≥6 g citric acid/L), and findings of the current study coincided with other similar studies [29]. However, pomegranate variety is a major factor affecting this characteristic. Different pomegranate varieties result in PAB with acidities from 4.6–20.2 g citric acid L^−1^ [29]. Statistically, the non-significance (*p* > 0.05) of all parameters at all cases is confirmed. In the present study, no significant differences were observed between PAB and initial pomegranate juice. The initial acidity (16 g citric acid L^−1^) that was detected in the present study, it is the usual (15.4−17.5 g citric acid L^−1^) reported in juices of pomegranate cv Wonderful [27,29].

### 3.2. Antioxidant Activity and Phenolic Compounds

Table 2 summarizes the effects of yeast and fermentation temperature on the antioxidant activity and phenolic content of PABs, in comparison with the characteristics of the initial pomegranate juice.

Pomegranate juice flavonoids have been extensively studied, with research suggesting that juice can contain amounts ranging from 45 to 636 mg QE L^−1^ [32,33]. Pomegranate juice used in this study appeared to fall within the expected values of flavonoid content (320.2 ± 4.5 mg QE L^−1^), a concentration which decreased after fermentation by approximately 70% at 15 °C and approximately 50% at 25 °C. Additionally, it can be concluded that both the effect of temperature and the effect of yeast were significant (*p* <0.05). These results are in accordance with previous studies reporting a reduction in TFC after fermentation at 25 °C from 30% to up to 63% [28].

Phenolic compounds contribute to the sensory characteristics of a product, as they affect parameters such as aroma, color, and flavor [34]. Pomegranate juice’s phenolic content (2470.1 ± 14.8 mg GAE L^−1^) is in accordance with other studies suggesting that it can contain amounts in a wide range of 458–7429 mg L^−1^ [32,33], and up to 3900 for Wonderful cultivar [29]. This concentration decreased after fermentation at 15 °C in the same way as flavonoids, namely by approximately 70%, whereas it decreased by approximately 65% at 25 °C. This decrease can be attributed to hydrolysis and oxidation reactions of polyphenols during fermentation, to condensation and polymerization reactions, as well as to adsorption of phenolics to the yeast cells [34,35]. Similarly, all effects were significant in all cases (*p* < 0.05). Previous studies reported a decrease in TPC of PAB after fermentation ranging from 7% up to 42% [28,29].

Free radical-scavenging activity (DPPH^●^) of the juice appeared to be equal to 17.6 mM TRE, with fermentations at 15 °C inducing a 15% decrease, and fermentations at 25 °C inducing a 10% decrease, approximately. The antioxidant activity of pomegranate juice depends greatly on the variety of pomegranate used [29]. The present study results are in accordance with those of a previous study with fermented PAB from Wonderful variety, reporting a decrease up to 16% [29]. In other PABs, which were produced using juices from other pomegranate varieties (Hicaz and Mollar de Elche), significantly smaller free radical-scavenging activities were reported [29,30]. It is possible that the decrease in free radical-scavenging activity observed after fermentation is due to oxidation reactions that took place between phenolics and/or other molecules [28]. It has been proved that fermented fruit juice production procedures can potentially alter the antioxidant activity of the product [36,37,38]. Temperature significantly affected the antioxidant activity while the effect of yeast was not significant.

The effect of yeast on the phenolic content and free radical-scavenging activity has been vastly studied on wine. Results seem to be contradicting, with researchers supporting that the effect of yeast on these characteristics is significant [39,40,41,42], while others support that there is no significant effect [43,44].

Finally, as far as monomeric anthocyanins are concerned, initial juice and PAB appeared to contain relatively high amounts of monomeric anthocyanins. The juice contained approximately 105.2 mg Cy3GE L^−1^, while fermentation caused a decrease of approximately 30% at both temperatures. Many researchers have studied the anthocyanin content of pomegranate juice and its fermented counterpart, suggesting a reduction after fermentation, usually 50% [4,28] and 46% for Wonderful variety [29]. The decrease of monomeric anthocyanin content observed after fermentation is probably due to the polymerization of monomeric anthocyanins, as polymeric anthocyanins cannot be detected by the pH differential method due to the fact that they are resistant to color change with change in pH and absorb at both values [18,45]. Additionally, this decrease can be due to degradation reactions and interactions between anthocyanins and other phenolic compounds [46]. Especially, during fermentation, the above-mentioned phenomena may be attributed to the condensation of anthocyanins with acetaldehyde, to enzymes like β-glucosidases that degrade anthocyanins, and to direct oxidation of anthocyanins by O_2_ [29,38,47]. Statistically, all effects appeared to be significant.

### 3.3. Volatile Composition

The composition of volatile compounds of PAB fermented with different yeasts (M02, SN9, and WB06) is presented in Table 3. A total of 30 different volatile compounds were identified, with the produced PABs containing 15 esters, 4 organic acids, 8 alcohols, and 3 terpenes, at concentrations that varied between samples. The most dominant compound group was found to be alcohols, followed by esters, organic acids, and terpenes, with the exception of fermentations with WB06 at 15 °C, where the concentration of esters was higher than that of alcohols. The majority of identified compounds are verified by literature and other studies on pomegranate and its juice/beverages [11,48,49,50]. However, there are many factors that can affect the concentration of these compounds, such as the fruit genotype and composition, the level of maturity at harvest, environmental and storage conditions, winemaking techniques, etc. [51].

The volatile analysis of the commercial pomegranate juice revealed a very low aromatic profile (mainly ethyl acetate and some alcohols were detected), as was already known by previous studies [52]. Industrial processing (including pasteurization) alters the volatile profile of juices, and therefore the majority of volatile compounds present in fresh juice are absent in commercial juices [49]. Therefore, it can be easily concluded that the majority of the compounds presented in Table 3 are a result of yeast metabolism and fermentation.

#### 3.3.1. Esters

Esters are compounds that can be found in fruits and vegetables and are significant aroma constituents responsible for these products’ fruity notes. Ethyl acetate is one of the most known aromatic compounds of fruit and vegetables belonging in this group and is thought to present a sweet-fruity odor. Specifically, studies have reported the presence of ethyl acetate in pomegranate juice [52], while the high intensity of pomegranate berry and fruity notes are attributed to esters (mainly ethyl acetate and octyl acetate) [11]. In the present study, ethyl acetate was the second most abundant ester, and it was detected in all samples. In general, higher concentrations were observed at lower temperatures. It was also detected in commercial juice but in relatively low concentrations (0.1 mg L^−1^). Ethyl propanoate and ethyl hexanoate have also been previously reported in pomegranate juice [53], compounds that were found to deplete after pasteurization. Both esters were detected in the PABs, however, ethyl hexanoate in higher concentrations as a result of the action of yeasts. Ethyl butanoate is a compound that has been previously found in commercial pomegranate products at high relative percentages [49]. However, it has not been listed as an important volatile in pomegranate [54]. In the present study, no significant differences were observed either between yeasts or temperature used, in the concentration of ethyl butanoate, ranging from 0.25 to 0.65 mg L^−1^. 3-methylbutyl acetate and 2-methylbutyl acetate were detected in all samples, with the first detected in higher concentrations. Both of them have been isolated from fresh pomegranate juice and are thought to contribute towards a fruity aroma [49,55]. Ethyl octanoate was the ester with the highest concentration in all samples. In general, the highest concentrations were detected at lower temperatures. Regarding the effect of yeast, WB06 resulted in higher concentrations followed by SN9 and M02. Our results are in accordance with previous studies that also found ethyl octanoate to be the most abundant compound in pomegranate wines, responsible for fruity, green, and citrus aroma [11]. 2-phenylethyl acetate, detected in all samples but at higher concentrations by WB06, is characterized as key volatile of pomegranate arils, providing a flowery, fruity, and cooked apple aroma [56]. Ethyl phenylacetate (fruit and sweet aroma), ethyl dodecanoate, and ethyl hexadecanoate (wax aroma) were identified in low concentrations in all samples, and they are usually found in pomegranate wines and vinegars [57]. Ethyl 9-decanoate (detected in all samples at 15 °C), and ethyl decanoate (detected in all samples), have not been reported in pomegranate products before. However, they have been reported in prickly pear wines [58]. In general, the PAB that were produced by WB06 yeast, presented the highest concentrations of esters in both fermentation temperatures.

#### 3.3.2. Organic Acids

Organic acids in beverages play an important role as far as flavor and taste are concerned. Firstly, they contribute to the development of sourness, while some of them possess their own characteristic flavor or aroma [59]. In wines, they are considered to contribute mainly to the complexity of aroma at concentrations not higher than their threshold values [60,61]. In the present study, four acids were detected, namely octanoic, decanoic, dodecanoic, and hexadecanoic acid. Octanoic and decanoic acids were detected in all samples, and they were the acids with the highest concentrations, as it was also reported in wines and cider [14,62]. Specifically, octanoic and decanoic acids have been identified in pomegranate juice [49,56] and wine [4,11]. Dodecanoic acid, detected only in M02 and WB06 PABs, derives from pomegranate seeds [63], and it has been found in small amounts in pomegranate juice and in higher concentrations in pomegranate wine and vinegar [57]. Hexadecanoic acid is considered to be the most common saturated fatty acid in plants and has been related to the crystalline structure of plant cell membranes [64]. However, in the present study, it was detected only in SN9 and M02 samples at low concentrations.

#### 3.3.3. Alcohols

Alcohols play an important role in the aromatic profile of fermented beverages. However, they are generally considered to have rather unpleasant odors and therefore, it is believed that they contribute more to the intensity of the odor of alcoholic beverages like wines and, therefore, to its quality [60]. 2-Phenylethanol is one of the few fusel alcohols described with a pleasant odor as old rose [60,65], which was detected in all PABs. A reduction in its content at low temperatures (15 °C) was observed, which is in accordance with other studies [66,67]. It is considered a key volatile of pomegranate aroma, accounting for almost 40% of total bound aroma compounds [68]. Another volatile found in almost all PABs was 1-hexanol and its presence is mainly correlated with pasteurized pomegranate products [49,52]. Similarly, 2-ethyl−1-hexanol, a compound with floral attributes, has been found in pomegranate juice [54]. In the present study, it was detected in M02 PAB and at SN9 PAB fermented at 25 °C. The alcohol with the highest concentration in all PABs was 3-methyl−1-butanol, who is responsible for a whiskey, malt, burnt aroma. It has also been found in the stem peels of pomegranates [48]. However, in the present study, its presence is mainly due to the action of yeasts during fermentation. 2,3-butanediol (fruit, onion aroma) is a compound that has previously been reported in commercial pomegranate juices [49] and laboratory-scale pomegranate wines [69] and is considered to be a secondary product of pyruvic acid during glyceropyruvic fermentations [26]. In the present study, it was detected in higher concentrations in PAB produced by SN9 yeast. Finally, (Z)-3-hexen−1-ol (grass aroma) has been reported as one of the major volatile compounds of pomegranate seeds [70] and was also found in pomegranate juices [50]. It was detected in all PABs at similar concentrations.

#### 3.3.4. Terpenes

Terpenes are compounds that are present in fruit juices naturally, partly in the form of glycosides, which can be hydrolyzed by enzymes or chemically. The processing of juices (e.g., heat treatment) can accelerate these phenomena and therefore alter the terpene profile of these products [71]. Eucalyptol (mint, sweet aroma) is a component that occurs naturally in a number of aromatic plants. As far as pomegranate is concerned, previous studies suggested that it can be found in both fresh pomegranate juice [50,55] and fermented pomegranate beverages [72]. In the present study, it was detected at low concentrations in PABs produced by M02 and SN9 yeasts. α-Terpineol, a monoterpene, is a compound found in pomegranate juice [2], contributing to the fruity aroma of products [68]. α-Terpineol was detected only in PAB produced by SN9 at 25 °C. It has also been detected in wines [22], however, due to its high perception threshold (0.4 mg L^−1^), it has very little olfactory impact on pomegranate beverages and wines [73]. Finally, trans-nerolidol that it was detected in all samples fermented at 25 °C, it is a terpene that occurs naturally in the essential oils of many plant and flower types, such as neroli, ginger, hemp, and lemon grass.

#### 3.3.5. Odor Activity Value of Aroma-Related Compounds

The odor activity values (OAVs), a marker of the influence on the aroma of individual volatile molecules, for each compound (which is higher than 1) is presented in Table 4. In general, compounds with OAV >1 are considered as odor-active compounds. As can be seen, the aroma-related compounds with OAV >1 belong mainly to esters and only two in alcohols. These results reveal the possible fruity character of all PABs produced in the present study. As in the case of concentrations, no significant differences were reported between the different temperatures and yeast strains.

#### 3.3.6. Chemometrics

**Figure 2:**
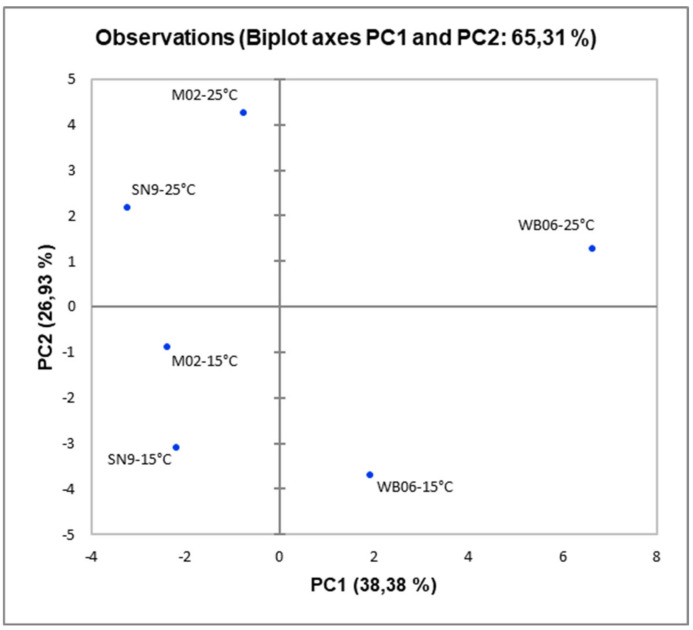
Principal component analysis (PCA) plot of minor volatiles isolated by pomegranate alcoholic beverages fermented at 15 °C and 25 °C by different yeasts (M02, SN9, WB06).

The PCA algorithm, applied to HS-SPME GC/MS data, showed that the fermentation temperature affected significantly volatile composition (Figure 2), since wines produced at 25 °C were placed at the upper side of the plot and wines produced at 15 °C were placed at the bottom. PAB produced by WB06 yeast contained compounds in amounts that correlated positively mostly to PC1, whereas M02 yeast resulted in compounds correlated mostly to PC2 for both fermentation temperatures. Consistently, by means of the PC1, it can establish a clear differentiation among the PAB obtained by fermenting pomegranate juice with different microorganisms. More specifically, PABs with M02 and SN9 are placed on the left side of the plot (negative values of PC1), while the PAB produced by WB06 (in both temperatures) are placed on the right side of the plot (positive values of PC1), characterized by the higher amount of alcohols and esters. On the other hand, according to the PC2, a clear distinction was observed between the PAB produced at different fermentation temperatures. The PAB produced at 25 °C, located at the upper side of the plot (positive values of PC2), while those produced at 15 °C, located at the lower side of the plot (negative values of PC2).

## 4. Conclusions

The present study clearly showed that the yeast strain and fermentation temperature are two of the most important factors that affect the characteristics of the final PABs. Different temperatures resulted in similar pH values and ethanol, glycerol, and acidity content, while significantly affected reducing sugars and antioxidant activities. More specifically, lower temperatures led to higher reducing sugars and lower antioxidant activities and phenolics, probably due to the higher fermentation time. In addition, it affected the volatile profile of final products. In general, all samples presented high free radical-scavenging activities, which is important in such products. Regarding the effect of microorganisms employed for the production of PAB, the selection of the most suitable yeast is tightly related to the nature and characteristics of the material used and the desired characteristics of the final product. In cases of high sugar content and high final alcoholic strength, it appears that SN9 yeast is the most suitable, while, in cases of low initial sugar content, WB06 and M02 yeasts seem better candidates. Finally, as far as the aroma profile (analyzed by GC/MS) of PABs is concerned, fermentation temperature is a factor that can cause distinctive profiles for all the yeasts studied, whereas among the yeasts, WB06 is the one that seems to differentiate. These results are considered very promising for the design and development of novel low alcohol products with increased functional characteristics and the appropriate selection of fermentation conditions. However, more studies are needed to evaluate the stability of functional compounds during storage and the possible inclusion of probiotic cells in combination with yeasts.

## Figures and Tables

**Table 1:** Effect of yeast and fermentation temperature on the physicochemical characteristics of pomegranate alcoholic beverages.

Analyses	Yeast Strain	Fermentation Temperature		Significance of Effect		
		15 °C	25 °C	Temp.	Yeast	Comb.
Reducing sugars (g D-glucose L^−1^)	PJ	128.4 ± 0.1		***	***	***
	M02	5.1 ± 0.3^b,A^	4.0 ± 0.1^b,B^			
	SN9	4.4 ± 0.2^c,A^	4.0 ± 0.2^b,B^			
	WB06	10.5 ± 0.2^a,A^	4.7 ± 0.1^a,B^			
Ethanol (% v/v)	PJ	-		ns	*	*
	M02	6.9 ± 0.1^a,A^	6.5 ± 0.2^a,A^			
	SN9	6.8 ± 0.1^a,A^	7.0 ± 0.1^a,A^			
	WB06	5.6 ± 0.2^b,A^	6.6 ± 0.1^a,B^			
Glycerol (g L^−1^)	PJ	-		**	**	**
	M02	5.75 ± 0.07^a,A^	5.65 ± 0.64^a,A^			
	SN9	5.05 ± 0.07^b,A^	6.05 ± 0.50^a,B^			
	WB06	2.65 ± 0.07^c,B^	5.80 ± 0.57^a,A^			
pH	PJ	3.12 ± 0.01^a,A^		***	ns	ns
	M02	3.11 ± 0.01^a,A^	3.07 ± 0.01^b,B^			
	SN9	3.12 ± 0.01^a,A^	3.06 ± 0.01^b,B^			
	WB06	3.10 ± 0.01^a,A^	3.05 ± 0.01^b,B^			
Volatile acidity (g acetic acid L^−1^)	PJ	0.11 ± 0.01^a,A^		*	***	ns
	M02	0.76 ± 0.05^c,B^	0.69 ± 0.04^b,B^			
	SN9	0.85 ± 0.05^c,B^	0.84 ± 0.02^c,B^			
	WB06	0.33 ± 0.01^b,B^	0.21 ± 0.01^a,A^			
Titratable acidity (g citric acid L^−1^)	PJ	16.0 ± 0.1^a,A^		ns	ns	ns
	M02	16.4 ± 0.6^a,A^	17.0 ± 0.2^a,A^			
	SN9	16.6 ± 0.4^a,A^	16.5 ± 0.6^a,A^			
	WB06	16.2 ± 0.3^a,A^	16.8 ± 0.5^a,A^			

^abcd^ Different letters at the same parameter and temperature (vertically–effect of yeast) indicate significant differences between means (*p* < 0.05). ^ABC^ Different letters at the same parameter and different temperature (horizontally–effect of temperature) indicate significant differences between means (*p* < 0.05). PJ, pomegranate juice; ns: not significant (*p* > 0.05); *: *p* < 0.05; **: *p* < 0.01; ***: *p* < 0.001; Temp, temperature; Comb, combined effect.

**Table 2:** Effect of yeast and fermentation temperature on the antioxidant activity and phenolic compounds of pomegranate alcoholic beverages.

Analyses	Yeast Strain	Fermentation Temperature		Significance of Effect		
		15 °C	25 °C	Temp.	Yeast	Comb.
Total flavonoid content (mg QE L^−1^)	PJ	320.2 ± 4.5^a,A^		***	***	ns
	M02	106.7 ± 26.4^b,C^	174.5 ± 19.4^b,B^			
	SN9	78.0 ± 34.3^b,C^	148.0 ± 16.4^c,B^			
	WB06	96.2 ± 27.8^b,C^	173.0 ± 11.7^b,B^			
Total phenolic content (mg GAE L^−1^)	PJ	2470.1 ± 14.8^a,A^		ns	ns	**
	M02	926.9 ± 357.8^b,B^	817.5 ± 98.4^c,B^			
	SN9	807.2 ± 385.1^b,B^	834.2 ± 86.8^c,B^			
	WB06	703.8 ± 69.2^b,C^	1027.3 ± 197.8^b,B^			
DPPH^●^ (mM TRE)	PJ	17.6 ± 0.1^a,A^		**	ns	ns
	M02	15.5 ± 1.4^b,A^	15.6 ± 1.5^b,A^			
	SN9	14.4 ± 1.1^b,C^	15.7 ± 1.0^b,B^			
	WB06	15.3 ± 0.9^b,C^	16.1 ± 0.5^b,B^			
Total monomeric anthocyanin content (mg Cy3GE L^−1^)	PJ	105.2 ± 0.1^a,A^		***	***	***
	M02	75.0 ± 3.4^b,C^	78.2 ± 2.1^b,B^			
	SN9	70.4 ± 1.1^c,C^	78.7 ± 2.0^b,B^			
	WB06	74.1 ± 0.7^b,B^	69.6 ± 2.6^c,C^			

^abc^ Different letters at the same parameter and temperature (vertically–effect of yeast) indicate significant differences between means (*p* < 0.05). ^ABC^ Different letters at the same parameter and different temperature (horizontally–effect of temperature) indicate significant differences between means (*p* < 0.05). PJ, pomegranate juice; QE, quercetin equivalents; GAE, gallic acid equivalents; TRE: trolox equivalents; Cy3GE, cyanidin-3-glucoside equivalents; ns, not significant (*p* > 0.05); *: *p* < 0.05; **: *p* < 0.01; ***: *p* < 0.001; Temp, temperature; Comb, combined effect.

**Table 3:** Effect of yeast and fermentation temperature on aroma-related compounds (mg L^-1^) of pomegranate alcoholic beverages as detected by GC/MS analysis.

Compound	Yeast Strain					
	M02		SN9		WB06	
	15 ^°^C	25 ^°^C	15 ^°^C	25 ^°^C	15 ^°^C	25 ^°^C
**Esters**						
ethyl acetate	3.14 ± 0.23	3.10 ± 0.99	5.90 ± 2.12	3.63 ± 1.10	3.40 ± 0.15	3.16 ± 0.91
ethyl propanoate	0.03 ± 0.04	0.15 ± 0.07	0.05 ± 0.07	0.20 ± 0.00	0.20 ± 0.00	Nd
ethyl butanoate	0.47 ± 0.07	0.30 ± 0.00	0.65 ± 0.49	0.25 ± 0.07	0.46 ± 0.08	0.45 ± 0.07
3-methylbutyl acetate	1.06 ± 0.02	2.00 ± 0.85	1.17 ± 0.18	1.63 ± 0.32	1.60 ± 0.56	2.23 ± 0.04
2-methylbutyl acetate	0.17 ± 0.01	0.23 ± 0.07	0.08 ± 0.11	0.22 ± 0.02	0.11 ± 0.11	0.13 ± 0.04
ethyl hexanoate	2.42 ± 0.05	2.06 ± 1.05	3.45 ± 0.92	3.00 ± 0.42	4.20 ± 0.57	7.99 ± 6.38
hexyl acetate	Nd	0.07 ± 0.03	Nd	0.40 ± 0.40	Nd	Nd
ethyl octanoate	3.90 ± 0.00^ab^	3.15 ± 1.48^a^	6.15 ± 0.78^ab^	4.58 ± 1.39^ab^	13.45 ± 2.19^b^	8.75 ± 4.03^ab^
ethyl phenylacetate	0.08 ± 0.04	0.11 ± 0.06	0.10 ± 0.00	0.09 ± 0.03	Nd	0.27 ± 0.19
2-phenylethyl acetate	0.23 ± 0.25	0.70 ± 0.44	0.54 ± 0.08	0.53 ± 0.32	1.14 ± 0.40	3.14 ± 1.89
ethyl 9-decanoate	0.25 ± 0.01	Nd	0.30 ± 0.28	Nd	0.20 ± 0.14	Nd
ethyl decanoate	1.10 ± 0.03	1.90 ± 1.13	2.06 ± 0.08	0.55 ± 0.07	6.15 ± 3.04	3.30 ± 1.27
ethyl dodecanoate	1.18 ± 0.54	1.62 ± 1.30	0.70 ± 0.00	0.18 ± 0.03	3.10 ± 1.13	2.74 ± 1.36
ethyl tetradecanoate	0.24 ± 0.20	0.50 ± 0.42	0.10 ± 0.00	0.08 ± 0.04	0.25 ± 0.07	0.15 ± 0.07
ethyl hexadecanoate	0.32 ± 0.31	1.09 ± 1.15	0.35 ± 0.07	0.29 ± 0.16	0.35 ± 0.21	0.70 ± 0.42
**Total esters**	**14.58 ± 1.46**	**16.98 ± 8.90**	**21.60 ± 4.97**	**15.61 ± 8.90**	**34.59 ± 5.99**	**33.00 ± 14.79**
**Organic acids**						
octanoic acid	3.89 ± 1.15	3.55 ± 2.47	5.75 ± 0.92	1.97 ± 0.23	7.75 ± 3.18	3.70 ± 0.28
decanoic acid	2.05 ± 1.06	2.10 ± 1.70	2.60 ± 0.57	0.55 ± 0.55	5.85 ± 4.17	5.35 ± 2.76
dodecanoic acid	0.52 ± 0.52	0.39 ± 0.39	Nd	Nd	0.59 ± 0.23	0.28 ± 0.28
hexadecanoic acid	0.06 ± 0.06	8.23 ± 8.23	Nd	0.03 ± 0.03	Nd	Nd
**Total organic acids**	**6.51 ± 2.84**	**14.26 ± 14.26**	**8.35 ± 1.48**	**2.54 ± 0.91**	**14.19 ± 7.59**	**9.33 ± 2.08**
**Alcohols**						
methyl-1-propanol	0.17 ± 0.23	0.50 ± 0.28	0.17 ± 0.17	0.28 ± 0.11	0.19 ± 0.19	0.44 ± 0.06
3-methyl-1-butanol	10.87 ± 0.57	15.83 ± 5.62	15.63 ± 5.05	14.17 ± 1.74	17.10 ± 2.03	62.08 ± 30.30
2-methyl-1-butanol	4.20 ± 0.28	7.74 ± 2.60	3.96 ± 0.93	3.70 ± 0.71	5.18 ± 0.69	8.49 ± 1.71
2,3-butanediol	Nd	0.85 ± 0.85	0.70 ± 0.70	0.20 ± 0.20	0.03 ± 0.03	Nd
(Z)-3-hexen-1-ol	0.17 ± 0.01	0.16 ± 0.08	0.15 ± 0.07	0.18 ± 0.05	0.10 ± 0.10	0.12 ± 0.03
1-hexanol	0.13 ± 0.01	0.18 ± 0.03	Nd	0.12 ± 0.06	0.05 ± 0.05	3.73 ± 3.73
2-ethyl-1-hexanol	0.12 ± 0.03	0.15 ± 0.07	Nd	0.05 ± 0.05	Nd	Nd
2-phenylethanol	2.25 ± 0.91	7.00 ± 3.54	3.50 ± 1.98	4.20 ± 0.14	9.40 ± 4.67	32.15 ± 26.23
**Total alcohols**	**17.89 ± 0.99**	**32.40 ± 5.09**	**24.11 ± 6.78**	**22.90 ± 0.60**	**32.03 ± 2.81**	**107.01 ± 63.46**
**Terpenes**						
eucalyptol	0.13 ± 0.00	0.12 ± 0.07	0.20 ± 0.00	0.11 ± 0.11	Nd	Nd
α-terpineol	Nd	Nd	Nd	0.10 ± 0.10	Nd	Nd
trans-nerolidol	Nd	0.15 ± 0.07^a^	Nd	0.14 ± 0.02^a^	Nd	0.62 ± 0.31^b^
**Total terpenes**	**0.13 ± 0.01**	**0.27 ± 0.14**	**0.20 ± 0.00**	**0.35 ± 0.28**	**Nd**	**0.62 ± 0.31**

^ab^ Different letters indicate significant differences between means (*p* < 0.05); Nd, not detected; The concentration of each group of compounds is presented in bold.

**Table 4:** Aroma compounds quantified in PABs with odor activity value (OAV) > 1, perception threshold, and aroma descriptions.

Compound	Threshold(mg L^−1^)	Odor Description^a^	Yeast Strain					
			M02		SN9		WB06	
			15 ^o^C	25 ^o^C	15 ^o^C	25 ^o^C	15 ^o^C	25 ^o^C
ethyl butanoate	0.4^a^	Strawberry, apple, banana	1.2 ± 0.2	<1.0	1.6 ± 1.2	<1.0	1.1 ± 0.2	1.1 ± 0.2
3-methylbutyl acetate	0.16^a^	Banana, fruity, sweet	6.6 ± 0.1	12.5 ± 5.3	7.3 ± 1.1	10.2 ± 2.0	10.0 ± 3.5	13.9 ± 0.3
ethyl hexanoate	0.08^a^	Fruity, green apple, banana, brandy, wine-like	30.2 ± 0.6	25.8 ± 13.1	43.1 ± 11.5	37.5 ± 5.3	52.5 ± 7.1	99.9 ± 79.7
ethyl octanoate	0.58^a^	Sweet, floral, fruity, banana, pear, brandy	6.7 ± 0.0	5.4 ± 2.6	10.6 ± 1.3	7.9 ± 2.4	23.2 ± 3.8	15.1 ± 6.9
2-phenylethyl acetate	1.8^a^	Flowery	<1.0	<1.0	<1.0	<1.0	<1.0	1.7 ± 1.0
ethyl 9-decanoate	0.1^b^	-	2.5 ± 0.1	Nd	3.0 ± 2.8	Nd	2.0 ± 1.4	Nd
ethyl decanoate	0.5^a^	Brandy, fruity, grape	2.2 ± 0.1	3.8 ± 2.3	4.1 ± 0.2	1.1 ± 0.1	12.3 ± 6.1	6.6 ± 2.5
ethyl dodecanoate	1.5^b^	-	<1.0	1.1 ± 0.9	<1.0	<1.0	2.1 ± 0.8	1.8 ± 0.9
3-methyl−1-butanol	60^a^	Solvent	<1.0	<1.0	<1.0	<1.0	<1.0	1.0 ± 0.5
1-hexanol	1.1^a^	Herbaceous, grass, woody	<1.0	<1.0	Nd	<1.0	<1.0	3.4 ± 3.4

^a^ [74]; ^b^ [75]; Nd, not detected.
